# Effect of modulated masking on electrophysiological and behavioral measures

**DOI:** 10.1590/2317-1782/e20230339en

**Published:** 2025-02-03

**Authors:** Mônyka Ferreira Borges Rocha, Karina Paes Advíncula, Danielle Samara Bandeira Duarte, Pedro de Lemos Menezes

**Affiliations:** 1 Programa de Pós-graduação em Biotecnologia – RENORBIO, Universidade Federal de Alagoas – UFAL - Maceió (AL), Brasil.; 2 Programa de Pós-graduação em Saúde da Comunicação Humana, Departamento de Fonoaudiologia, Universidade Federal de Pernambuco – UFPE - Recife (PE), Brasil.; 3 Programa de Pós-graduação em Saúde da Comunicação Humana, Universidade Federal de Pernambuco – UFPE - Recife (PE), Brasil.; 4 Departamento de Fonoaudiologia, Universidade Estadual de Ciências da Saúde de Alagoas – UNCISAL - Maceió (AL), Brasil.

**Keywords:** Electrophysiology, Evoked Potentials Auditory, Speech Perception, Psychoacoustics, Perceptual Masking, Hearing

## Abstract

**Purpose:**

To analyze the Benefit of Modulated Masking (BMM) in electrophysiological and behavioral measurements in young and adult normal-hearing individuals.

**Methods:**

Observational and cross-sectional analytical study, with a final research sample consisted of 40 participants, 20 individuals aged 18 to 30 years (young adults) and 20 individuals aged 31 to 50 years (adults), to carry out behavioral assessment (Sentence recognition test in the presence of stable and modulated noise) and electrophysiological (Cortical Auditory Evoked Potential) for BMM investigation. The results were analyzed using the paired t-test and ANOVA for repeated measures, applied by the Bonferroni post-hoc test (p-value <0.05).

**Results:**

Less interference from modulated noise was identified in the latency and amplitude measurements of cortical components, generating a significant reduction in P1 latency and an increase in P2 amplitude in both groups of participants. Stable noise generated higher electrophysiological and behavioral thresholds compared to modulated noise. A greater magnitude of BMM was observed in the young-adult group.

**Conclusion:**

In both groups of participants, less interference from modulated noise was identified in the encoding time of the neural auditory response and in the process of neural discrimination of speech. Furthermore, behavioral and electrophysiological thresholds were typically higher in stable noise when compared to modulated noise, pointing to a correspondence between BMM measurements between hearing domains. The magnitude of the higher BMM in the young-adult group, especially in the electrophysiological domain, suggests a greater temporal resolution ability in younger individuals.

## INTRODUCTION

The use of Long Latency Auditory Evoked Potentials (LLAEP), such as Cortical Auditory Evoked Potential (CAEP), has grown significantly in clinical audiological practice for diagnosis and auditory monitoring, as well as in research for the development of new studies and methodologies related to Central Auditory Processing (CAP) in different populations^(1)^.

The CAEP allows the assessment of the neuroelectrical activity of the auditory cortex, providing important biological data on human auditory processing. It can be easily applied, as it is independent of the patient's response, attention, and interaction, and is performed with minimal discomfort^(2)^.

One of the CAP skills that can be studied through CAEP is the individual's Temporal Auditory Processing (TAP) capacity, which integrates important functions in communication, especially for understanding speech^(3)^. TAP involves decoding of temporal aspects of hearing, enabling the individual to process subtle changes in sound or speech over time and to discriminate between the physical characteristics of time, frequency and intensity of the acoustic signal^(4)^.

The phenomenon known as Masking Release, referred to in Portuguese as Benefit of Modulated Masking – BMM^(5)^, is an effect related to TAP that involves the identification of audible signals in sound or speech despite fluctuations in simultaneous background noise^(6)^. In other words, BMM occurs when temporal fluctuations in the masking noise allow audible signals from the target sound or speech to be heard, improving the recognition performance of the target signal^(7)^.

Behavioral hearing measures, through psychoacoustic tests, were initially used to investigate BMM^(5,8-10)^. These tests found an improvement in the speech recognition threshold when exposed with modulated masking, compared to steady masking, and linked this performance with the individual's temporal processing capacity.

Research investigating BMM in the field of auditory electrophysiology and studying the effect of masking modulation on the behavior of objective cortical measures, such as CAEP, has shown that these tests have the potential to assess TAP skills. Additionally, they present objective results for evaluation individuals unable to provide reliable behavioral responses^(6,11-13)^.

The study of the BMM phenomenon in the electrophysiological and behavioral domains has advanced research on TAP abilities^(6)^ and established parameters for analyzing speech recognition in noisy environments^(13)^. However, the literature indicates that studies using electrophysiological measures to investigate BMM typically do not conduct parallel behavioral tests^(11,12)^.

It is assumed that by performing electrophysiological measurements alongside behavioral measurements, it is possible to determine whether these objective measures are predictive of auditory behavioral performance in the study of BMM and its effect on temporal auditory processing. Therefore, this study aims to analyze BMM in electrophysiological and behavioral measurements in young adults with normal hearing.

## METHODS

The research is an analytical, observational and cross-sectional study, conducted at the Audiology Laboratory of the Speech Therapy Department of the Federal University of Pernambuco, from August 2022 to June 2023. The research protocol follows Resolution N^o^. 466/2012 of the National Health Council (NHC) for studies with human subjects and was approved by the Ethics and Research Committee on Human Beings of the Federal University of Pernambuco, under opinion number 5,140,668.

Initially, 22 individuals aged 18 to 30 years (young adults) and 23 individuals aged 31 to 50 years (adults) participated in this research, recruited on the university campus after the research was publicized electronically. All participants were informed about the objectives and procedures necessary to carry out the study. After agreeing to participate, they signed two copies of the Free and Informed Consent Form (FICF).

Regarding the eligibility criteria for participation in the research, the inclusion criteria were individuals between 18 and 50 years of age without hearing loss. Individuals with a history of neurological and/or psychiatric diseases, cognitive deficits, malformations of the auricle and external auditory canal, as well as any type and/or degree of hearing loss, were excluded. To ensure that the research selection criteria were met, a detailed anamnesis with information on the participants' general and auditory health was conducted on a previously scheduled date. This included basic audiological exams (inspection of the external auditory canal, audiometry and immittance testing) and a cognitive screening test (Montreal Cognitive Assessment Test - MoCA)^(14)^.

The presence of alterations and/or malformations in the external and/or middle ear was ruled out by inspection of the external auditory canal, along with the immittance test (226 Hz probe). A type A tympanometric curve with both ipsilateral and contralateral reflexes was considered normal^(15,16)^. To confirm the normality of the tonal auditory thresholds in the audiometry test, the standard for normality was defined as a four-frequency pure-tone average of 500 Hz, 1000 Hz, 2000 Hz and 4000 Hz below 20 dB HL, in both ears^(17)^. For the cognitive screening (MoCA), a normal result was obtained with a score of ≥ 26 points, as suggested in the test itself^(14)^.

### Behavioral assessment

To investigate BMM in the behavioral domain, participants underwent a sentence recognition test in the presence of steady and modulated noise, using the 12 lists (each containing 20 sentences) of the HINT-Brazil test, recorded in the male voice of a native Brazilian speaker^(18)^. The test was performed to obtain the Sentence Recognition Threshold (SRT) in both noise conditions, which was considered the individual's behavioral threshold. The steady noise was presented at 65 dB SPL, while the modulated noise varied between intensities of 65- and 30-dB SPL with a modulation rate of 10 Hz^(8)^. The noise used (speech-shaped noise, or SSN) has a frequency spectrum envelope similar to that of the sentences used in the test. Modulated noise was produced by modifying the steady-state noise using a Tucker-Davis Technologies-RX6 (TDT-RX6) acoustic signal processor^(5,8)^. The presentation of masking noises followed a random order for each participant.

To perform the test, participants were seated in an armchair inside a soundproof booth and instructed to repeat the sentences in the presence of competitive background noise exactly as they heard them. The participants' responses were monitored and recorded simultaneously by the examiner positioned outside the booth, using MATLAB software (Matrix Laboratory®), version R2012a. The sentences and competitive background noise were sent via the TDT-RX6 and presented monaurally through supra-aural earphones (Sennheiser HD580) to the right ear. Each sentence from the HINT-Brazil was presented only once to the same participant, to eliminate learning bias, and the lists were chosen randomly. The test lasted approximately 40 minutes, with breaks being taken upon request by the subjects.

The correct answer for each sentence was determined based on its exact repetition; any change in the use of articles, verb conjugation, and inclusion or omission of words was considered errors. Given the importance of reproducibility in obtaining the SRT, three threshold measurements were performed for both noise conditions. The final SRT of each participant (for both types of noise) was obtained by averaging the three threshold measurements in dB SPL. The BMM was defined by the difference between the SRT in the presence of steady noise (used as a reference) and the SRT in the presence of modulated noise.

For the SRT research, the initial intensity used was higher than the expected recognition threshold, set at 60 dB SPL for the modulated noise condition and 70 dB SPL in the steady noise condition. The method for obtaining the SRT, using MATLAB software, involved a descending-ascending transformed type (two down, one up)^(19)^. For every two consecutive correct answers, the signal intensity decreased by 2 dB for the following sentence, while for each incorrect answer, the presentation intensity of the following sentence was increased by 2 dB. Each SRT was obtained after six reversals (increase or decrease in the intensity of the presented sentences) by calculating the average of the four final reversal levels (intensities).

### Electrophysiological assessment

To investigate BMM in the electrophysiological domain, participants underwent the Intelligent Hearing Systems (IHS) CAEP with synthetic speech stimulus, /ba/, in the presence of speech noise (SSN)^(20)^, presented simultaneously, with the noise in two conditions: steady and modulated. The /ba/ stimulus was presented in a modified waveform (rate of 24,414 Hz) to ensure compatibility with the digital signal of the Tucker-Davis Technologies-RX6 (TDT-RX6) processing platform and calibrated with reference to the dB SPL of a 1 kHz continuous tone, equivalent peak (dB SPLpe). To record the potentials, a recording system was synchronized between the IHS Smart EP and the TDT-RX6 using a time-event marker (“Trigger”) that coincided with the onset of each /ba/ stimulus. The speech stimulus /ba/ and noise were presented monaurally to the right ear via an electromagnetically shielded insert earphone (ER2) connected directly to the TDT-RX6. The /ba/ had a duration of 80 milliseconds (ms) and was presented at a fixed intensity of 65 dB SPL and a rate of 3.8 stimuli per second.

**Figure 1:**
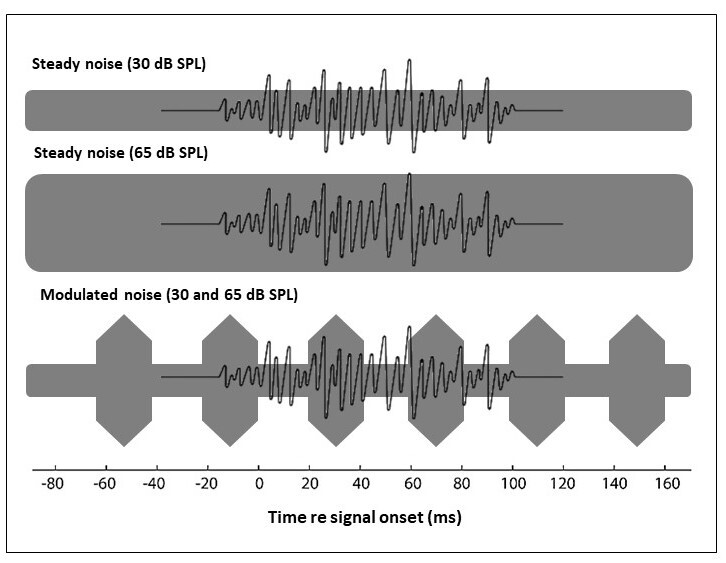
Illustration of the speech stimulus under the three masking conditions

For CAEP acquisition, the noise was presented simultaneously with the /ba/ stimulus in three different conditions: a) /ba/ and steady noise at an intensity of 30 dB SPL (weak steady noise); b) /ba/ and steady noise with an intensity of 65 dB SPL (strong steady noise); and c) /ba/ and noise modulated at 25 Hz at intensities of 30 and 65 dB SPL (Figure 1). The presentation of the different noise conditions was performed randomly for each participant.

Caption: Representation of the speech stimulus /ba/ (black wave) in the three noise conditions used in the electrophysiological test

Participants were seated in a reclining chair inside an acoustic booth, watching a video without audio, and were instructed not to fall asleep during the examination. The skin was cleaned with 70% alcohol and abrasive gel (NuPrep®) before the electrodes were placed. The electrodes were positioned in the following configurations: two negative polarity reference electrodes located of the right (A1) and left (A2) lobes; one positive polarity electrode placed on the vertex of the head (Cz); and one ground electrode positioned in the lower region of the forehead (Fpz). The eartips used in the insert earphones were disposable. The total duration of the examination was approximately one hour for each participant, with breaks being taken upon request.

The cortical potentials (P1, N1, and P2) were analyzed for their latency (in milliseconds, ms), amplitude (in microvolts, µV) and morphology under the three noise conditions. All CAEP tracings were analyzed individually and blindly by three evaluators with experience in electrophysiology, to identify and mark the potentials. The P1 component was defined as the first robust positive cortical wave occurring around 50 ms, the N1 component was analyzed as the through following to the P1 wave, which exhibited greater negativity, and the P2 response was identified as the most robust positive wave after N1.

The electrophysiological threshold in CAEP was also investigated for each participant, elicited by the speech stimulus /ba/ under conditions of strong steady noise and modulated noise. The threshold was determined by decreasing the intensity of the speech stimulus by 10 dB until the P1-N1-P2 complex disappeared and then increasing it by 5 dB until it reappeared. After obtaining the electrophysiological threshold, the magnitude of the BMM was measured for each subject based on the difference in decibels (dB SPLpe) between the steady and modulated masking conditions.

### Statistical analysis of the data

Statistical analysis was performed using the Statistical Package for the Social Sciences (SPSS) version 20.0. The results were expressed as statistical measures of mean, median, standard deviation and confidence interval (95%), presented in tabular format. The normality of the samples per group was verified using the Shapiro-Wilk test and the Hair et al.^(21)^ criterion for analysis of asymmetry (-2 and +2) and kurtosis (-7 and +7), with a normal distribution of the data observed. For inferential analysis of the cortical components in each noise group, ANOVA for repeated measures was used, followed by the Bonferroni post-hoc test (p-value <0.05). The paired t-test was used to analyze the electrophysiological and behavioral thresholds between the two noise groups, steady and modulated (p-value <0.05).

## RESULTS

A total of 45 individuals were initially recruited for the research, with 22 participants aged 18 to 30 years and 23 participants aged 31 to 50 years. Five participants were excluded from the final sample, two due occlusion of the external auditory canal, one due to thresholds compatible with mild hearing loss, and two for not attending the behavioral and electrophysiological examination on the scheduled date. As a result, 40 participants with hearing within pre-established normal criteria comprised the final sample of the study. Participants were divided into two groups according to age: the ’Young-Adult’ (ages 18 to 30 years old) and the ‘Adult’ group (ages 31 to 50 years old). The Young-Adult group consisted of 20 participants (15 women and 5 men), with an average age of 22.8 years, while the Adult group was made up of 20 participants (10 women and 10 men), with an average age of 37.7 years old.

**Figure 2:**
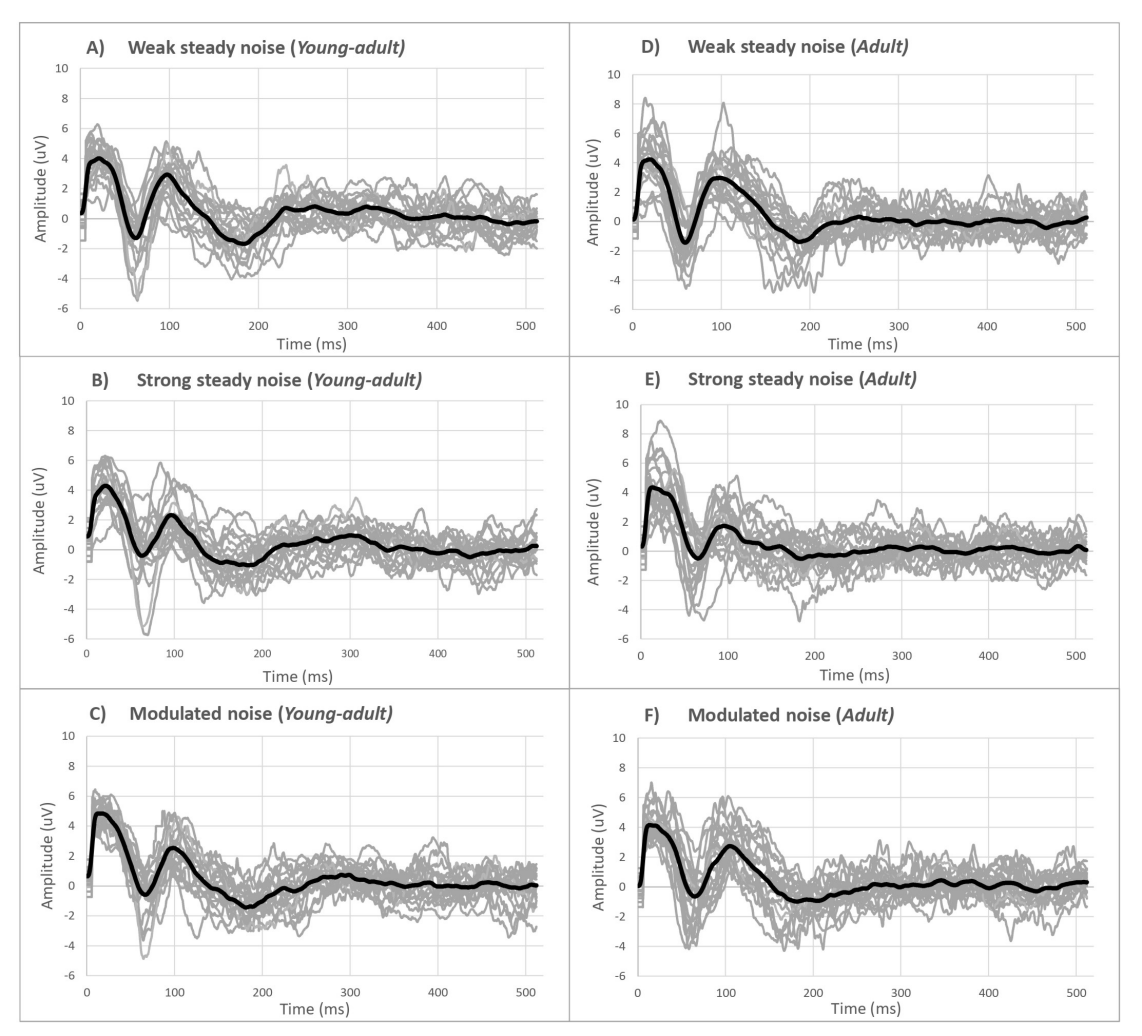
Grand averages of the CAEP waves for the three masking conditions and in each group of participants.

The grand average response of the CAEP waves to each type of masking noise, and between the two groups of participants, is shown in panels A – F of Figure 2. Individual traces are represented by light gray lines, while the mean responses of each group are indicated by bold dark lines. A similarity in the average cortical waves responses between the age groups can be observed. In the strong steady noise condition, the cortical waves show lower amplitudes, particularly in the P2 component (represented by the second positive peak) and in the N1 component (represented by the first negative valley), for both groups of participants (Figure 2B and E).

Table 1 presents the latency values ​​of the cortical components (P1, N1, P2), evoked by the speech stimulus /ba/ for each age group, in response to the three different types of masking noise, along with the comparison of the means obtained. It is possible to observe that for both the young adult and adult groups, the latency values ​​for the modulated noise condition were lower when compared to the strong steady noise.

**Table 1:** Comparison of the average latency of the P1, N1 and P2 components between the different noise conditions

**Latency**	**Weak steady noise**	**Strong steady noise**	**Modulated noise**	**ANOVA**
**(ms)**	**Mean ± SD**	**Mean ± SD**	**Mean ± SD**	**Post-hoc (Bonferroni)**
	**(CI _95%_)**	**(CI _95%_)**	**(CI _95%_)**	
		**Young-adult**		
Component				
P1	56.3 ± 11.5	68.9 ± 20.1	51.9 ± 8.0	**p 0.016 ^(a)^** *
	(50.8 - 6.7)	(59.4 - 78.3)	(48.1 - 55.6)	p 0.896 ^(b)^
				**p 0.003 ^(c)^** *
				
Component	109.6 ± 10.0	118.7 ± 23.2	116.6 ± 12.3	p 0.328 ^(a)^
N1	(104.9 - 114.3)	(107.8 - 129.6)	(110.8 - 122.4)	p 0.059 ^(b)^
				p 1.000 ^(c)^
				
Component	165.9 ± 16.1	174.9 ± 24.2	168.3 ± 12.9	p 0.521 ^(a)^
P2	(158.3 - 173.4)	(163.1 - 186.2)	(162.2 - 174.4)	p 1.000 ^(b)^
				p 0.488 ^(c)^
		**Adult**		
Component				
P1	56.4 ± 9.3	65.6 ± 10.3	54.8 ± 7.5	**p 0.024 ^(a)^** *
	(52.1 - 60.8)	(60.7 -70.5)	(51.3 - 58.4)	p 1.000 ^(b)^
				**p 0.002 ^(c)^** *
				
Component	104.6 ± 7.7	119.9 ± 21.5	116.7 ± 15.1	p 0.022 (a)
N1	(100.9 - 108.2)	(109.8 - 129.9)	(109.6 - 123.8)	p 0.008 (b)
				p 1.000 (c)
				
Component	163.8 ± 13.7	178.7 ± 27.4	173.2 ± 22.0	p 0.108 ^(a)^
P2	(157.4 - 170.3)	(165.9 - 191.6)	(162.8 - 183.4)	p 0.204 ^(b)^
				p 0.793 ^(c)^

* Statistically significant difference

^(a)^Comparison of means between weak and strong steady noise;

^(b)^Comparison of means between weak steady noise and modulated noise;

^(c)^Comparison of means between strong steady noise and modulated noise

Caption: ms = milliseconds; SD = Standard Deviation; CI _95%_ = 95% Confidence Interval

In the comparison test of latency measurements (ANOVA, post-hoc Bonferroni), it was found that the reduction of this measure with noise modulation was statistically significant (p<0.05) for the P1 cortical response in compared to the strong steady noise condition in both participant groups. There was no significant difference in latencies, that is, in the neural coding time between modulated and weak steady masking.

In Table 2, the amplitude values ​​of the cortical responses (P1, N1, P2) for the three masking conditions used are described for the two groups of participants, as well as the comparison of the means. It was observed that in young adult group, the mean amplitude values ​​of the cortical components were higher with modulated noise compared to strong steady noise.

**Table 2:** Comparison of the average amplitudes of the P1, N1 and P2 components between the different noise conditions

**Amplitude**	**Weak steady noise**	**Strong steady noise**	**Modulated noise**	**ANOVA**
**(µV)**	**Mean ± SD**	**Mean ± SD**	**Mean ± SD**	**Post-hoc (Bonferroni)**
	**(CI _95%_)**	**(CI _95%_)**	**(CI _95%_)**	
		**Young-adult**		
				
Component	5.7 ± 1.9	5.2 ± 1.9	5.8 ± 1.6	p 0.565 (a)
P1	(4.8 - 6.6)	(4.3 - 6.1)	(5.0 - 6.6)	p 1.000 (b)
				p 0.373 (c)
				
Component	5.1 ± 3.1	4.2 ± 3.2	4.4 ± 2.4	p 0.123 ^(a)^
N1	(3.6 - 6.6)	(2.6 - 5.7)	(3.2 - 5.5)	p 0.370 ^(b)^
				p 1.000 ^(c)^
				
Component	5.0 ± 4.1	3.3 ± 2.5	5.4 ± 2.4	**p 0.027 ^(a)^** *
P2	(3.0 - 6.9)	(2.1 - 4.5)	(4.2 - 6.5)	p 1.000 ^(b)^
				**p 0.001 ^(c)^** *
		**Adult**		
				
Component	5.3 ± 1.8	5.4 ± 2.0	5.4 ± 1.7	p 1.000 ^(b)^
P1	(4.4 - 6.2)	(4.4 - 6.3)	(4.6 - 6.3)	p 1.000 ^(b)^
				p 1.000 ^(b)^
				
				
Component	5.4 ± 2.3	4.1 ± 3.1	4.8 ± 2.4	p 0.055 ^(a)^
N1	(4.3 - 6.5)	(2.6 - 5.6)	(3.6 - 5.9)	p 0.368 ^(b)^
				p 0.690 ^(c)^
				
Component	4.9 ± 2.6	3.4 ± 2.0	5.2 ± 1.8	p 0.084 ^(a)^
P2	(3.6 - 6.1)	(2.5 - 4.4)	(4.4 - 6.1)	p 1.000 ^(b)^
				**p 0.006 ^(c)^** *

* Statistically significant difference

^(a)^Comparison of means between weak and strong steady noise;

^(b)^Comparison of means between weak steady noise and modulated noise;

^(c)^Comparison of means between strong steady noise and modulated noise

Caption: µV = microvolts; SD = Standard Deviation; CI _95%_ = 95% Confidence Interval

The comparative test (ANOVA, post-hoc Bonferroni) demonstrated that the increase in amplitude measurements in the noise modulation condition was statistically significant (p<0.05) in the cortical response of P2 compared to the strong steady noise condition. In the adult group, amplitude responses were greater in the N1 and P2 components in the face of masking modulated noise compared to strong steady noise, with a statistically significant difference (p<0.05) only in P2, similar to the younger group. As with latency responses, there were no significant differences in amplitudes, that is, in the magnitude of the neural response, between modulated and weak steady masking for both age groups.

In the research on electrophysiological and behavioral thresholds, the results shown in Table 3 indicate that, for both groups of participants, the masking modulation condition resulted in statistically lower thresholds (p<0.05; paired t test) compared to the steady masking condition in both domains of hearing.

**Table 3:** Description of BMM and electrophysiological and behavioral thresholds in the face of steady noise and modulated noise

	**Steady noise**	**Noise modulated**	**BMM**	**Test t paired**
(dB SPL)	Mean ± SD	Mean ± SD	Mean ± SD	p - value
	(CI _95%_)	(CI _95%_)	(CI _95%_)	
		
**Young-adult**				
				
**Electrophysiological threshold**	49.7 ± 6.3	41.2 ± 5.3	9.5 ± 4.6	p 0.000*
	(46.7 - 52.7)	(38.7 - 43.7)	(7.0 - 11.4)	
				
**Behavioral threshold**	59.1 ± 1.0	50.3 ± 1.8	8.7 ± 1.4	**p 0.000** *
	(58.6 - 59.6)	(49.4 - 51.2)	(8.1 - 9.4)	
		
**Adult**				
				
**Electrophysiological threshold**	52.5 ± 4.1	45.5 ± 3.5	6.7 ± 2.4	**p 0.000** *
	(50.5 - 54.4)	(43.8 - 47.1)	(5.6 - 7.9)	
				
**Behavioral threshold**	59.2 ± 1.0	51.0 ± 1.6	8.1 ± 1.3	**p 0.000** *
	(58.7 - 59.6)	(50.2 - 51.8)	(7.5 - 8.7)	

* Statistically significant difference

Caption: SD = Standard Deviation; CI _95%_ = 95% Confidence Interval; dB SPL = decibel sound pressure level; BMM = Benefit of Modulated Masking

**Figure 3:**
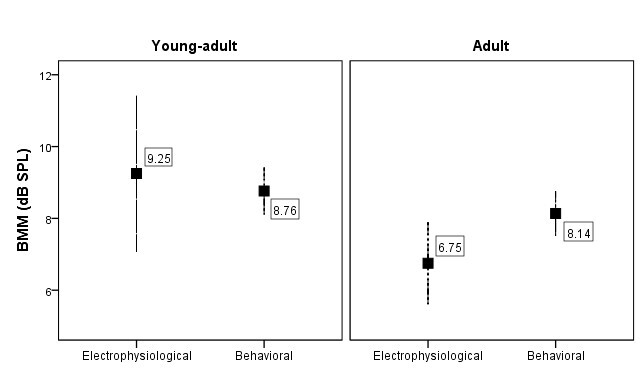
Benefit of electrophysiological and behavioral modulated masking (BMM) for the two groups of participants

Legend: Individual (light gray) and group average (black) CAEPs for Weak Steady Noise, Strong Steady Noise, and Modulated Noise, for each group of participants

This benefit, observed by the reduction of thresholds in response to noise modulation, is expressed by the BMM measurement, being greater in the young adult group across both hearing domains, as illustrated in Figure 3.

## DISCUSSION

The study of the effect of modulated noise on the detection of speech stimuli through CAEP allows the analysis of the benefit of modulation and its effect on the temporal processing of hearing in an objective manner. It highlights the importance of psychoacoustic assessment in order to investigate a prediction of this effect on auditory behavioral performance. Consequently, this study evaluated the effect of modulating masking noise with speech stimuli by comparing the electrophysiological and behavioral in young and adults with normal hearing.

The P1-N1-P2 cortical complex evoked by the speech stimulus /ba/, masked by steady and modulated noise, allowed the analysis of cortical responses related to the acoustic characteristics of sound processing at the thalamic level, the primary auditory cortex and association areas^(22)^. The application of CAEP with complex stimuli and under masking conditions favors the study of BMM, mainly due to the proximity of the generators of its potentials to the perception of sound^(23)^.

In this way, the latency and amplitude responses of the analyzed cortical components can reflect the perception of the acoustic characteristics of speech and determine the integrity of each individual's neural coding. Additionally, they can provide insights into the influence of noise on speech perception time and the magnitude of cortical activity in processing these complex signals^(24)^.

Based on the analysis of latency measurements, the present study observed greater interference from strong steady noise in the neural synchronism required for generating cortical potentials (P1-N1-P2) in both groups of participants, resulting in longer latencies in this masking condition (Table 1). Lesser interference in this neural synchronism, similar in both age groups, was statistically observed in the generation of the P1 component due to the decrease in noise intensity (weak steady noise) and its modulation. This finding demonstrates that, despite noise disturbance in speech perception, modulated noise has less interference in the coding time of the neural auditory response (represented by P1) when compared to strong steady noise, with no difference in neural coding time between modulated masking and weak steady noise.

Despite the expected delay in the latencies of cortical responses under noise conditions, studies also indicate less interference in neural processing time when this noise presents modulations, resulting in a systematic decrease in latency responses compared to steady noise^(6,12,13)^. The lower significant interference of the modulation expressed in the P1 component can be explained by the fact that this potential is related to the detection and coding of the frequency and temporal characteristics of the acoustic stimulus^(25)^, with these properties being better explored during the reduction of masking levels of the modulated noise, facilitating the encoding time of the speech signal^(7).^

Regarding amplitudes (Table 2), the results showed, similar to the latency results, less interference from modulated masking in these responses, resulting in larger amplitudes when compared to the strong steady noise condition, especially for the P2 component (p<0 .05), for both groups of participants. The morphology of the grand average waves confirms the greater interference of strong steady noise in cortical responses, as expressed by the smaller amplitudes of the second positive wave (P2) and the first negative trough (N1) (Figure 2B and E). These results corroborate studies that point to a decrease in the signal/noise ratio generated by the modulation in the intensity of the masking noise reflecting less interference from this masking in the magnitude of the neural responses expressed by the potential amplitudes^(6,26)^.

The lower significant interference generated by modulated noise in the P2 amplitude demonstrates that despite masking, the modulation condition still allows for better mobilization of neurons in the process of neural discrimination of the speech signal, with greater disturbance occurring in the steady noise condition^(27)^. Regarding the latency and amplitude measurements of the cortical components studied, there were no considerable differences in the responses in both groups of participants, indicating similar cortical auditory performance across the age groups studied.

The results of the electrophysiological and behavioral threshold research for both age groups (Table 3) corroborate findings in the literature that indicate that steady noise has a greater masking effect on the minimum thresholds for detecting a sound signal, generating typically higher thresholds compared to modulated noise in both hearing domains^(5,6,10)^. Although the average CAEP thresholds were lower than those recorded for behavioral thresholds, the difference was less than 10 dB, which aligns with other findings in the literature that observed comparatively lower cortical thresholds than behavioral thresholds by up to 10 dB^(28)^.

In the present research, the statistically lower thresholds in the presence of modulated noise in both hearing domains demonstrate that performing CAEP can be predictive in assessing auditory behavioral performance, confirming the benefit of noise modulation in the detection and perception of speech stimuli. The study between electrophysiological and behavioral thresholds has been highlighted in other research, showing a correlation between these measurements^(23,29)^, where CAEP has shown potential to evaluate temporal processing abilities and provide reliable equivalent results for detection thresholds of speech when faced with steady and modulated noises^(6,13)^.

The threshold difference between the two masking conditions (steady and modulated), interpreted as the BMM measurement, was comparably similar between the electrophysiological and behavioral domains in both groups of participants (Table 3), indicating a correspondence of this measure between the domains of hearing. The BMM value, particularly in the electrophysiological domain, was higher in the young-adult group (9.5 dB) compared to the adult group (6.7 dB), demonstrating a better use of noise modulation in younger individuals (Figure 3), suggests that younger individuals have a great capacity of the auditory system to perceive acoustic speech cues that are not masked in periods of time with lower noise intensity due to modulation^(5,30)^.

The magnitude of the BMM in the behavioral domain was approximately the same between the groups of participants, demonstrating little variation related to the age group studied in the psychoacoustic test. BMM research studies using behavioral tests in young people and adults found magnitudes of 8.6 dB and 7.3 dB, which are similar to the results found in the present research^(5,8)^.

Research on the effect of noise modulation on the auditory system suggests that the BMM response can be adopted as a representative measure of temporal resolution capacity^(6,31)^, since this ability refers to the identification of short temporal periods in response to two acoustic signals^(32)^. Thus, it is possible to consider that the presence of the BMM effect is related to the integrity of temporal processing of hearing in normal-hearing individuals.

The results of this research allowed for the analysis of BMM in the behavioral and electrophysiological domains of hearing, enabling a better understanding of the effect of noise modulation on temporal auditory processing, as well as its relationship with the temporal resolution ability. Although this research is restricted to the young and adult normal-hearing population, further studies with this age group, as well as with the elderly population, both with and without hearing loss, may contribute to a better understanding of BMM.

## CONCLUSION

The findings of the present research indicated that, in both groups of participants, there was less interference from modulated noise in the coding time of the neural auditory response (significant reduction in P1 latency) and less disturbance from modulated noise in the process of neural speech discrimination (significant increase in P2 amplitudes). Research into behavioral and electrophysiological thresholds showed that steady noise generated typically higher thresholds compared to modulated noise in both tests, pointing to a correspondence in the BMM measurement across the hearing domains. Young adults exhibited a higher BMM magnitude than adults, especially in the electrophysiological domain, which suggests a greater ability for temporal resolution in younger individuals.
