# Supplementary material for: Effect of modulated masking on electrophysiological and behavioral measures
Source: Codas. 2025 Feb 3;37(1):e20230339. doi: 10.1590/2317-1782/e20230339en (PMC11801197; doi:10.1590/2317-1782/e20230339en)
Supplement: Translation

# Translation: Efeito do mascaramento modulado em medidas eletrofisiológicas e comportamentais

**DOI:** 10.1590/2317-1782/e20230339pt

**Authors:** Mônyka Ferreira Borges Rocha, Karina Paes Advíncula, Danielle Samara Bandeira Duarte, Pedro de Lemos Menezes

**Affiliations:** 1 Programa de Pós-graduação em Biotecnologia – RENORBIO, Universidade Federal de Alagoas – UFAL - Maceió (AL), Brasil.; 2 Programa de Pós-graduação em Saúde da Comunicação Humana, Departamento de Fonoaudiologia, Universidade Federal de Pernambuco – UFPE - Recife (PE), Brasil.; 3 Programa de Pós-graduação em Saúde da Comunicação Humana, Universidade Federal de Pernambuco – UFPE - Recife (PE), Brasil.; 4 Departamento de Fonoaudiologia, Universidade Estadual de Ciências da Saúde de Alagoas – UNCISAL - Maceió (AL), Brasil.

**Keywords:** Eletrofisiologia, Potenciais Evocados Auditivos, Percepção da Fala, Psicoacústica, Mascaramento Perceptivo, Audição

## Abstract

**Objetivo:**

Analisar o Benefício do Mascaramento Modulado (BMM) em medidas eletrofisiológicas e comportamentais em indivíduos jovens e adultos normouvintes.

**Método:**

Estudo analítico observacional e transversal, com amostra final da pesquisa composta por 40 participantes, sendo 20 indivíduos de 18 a 30 anos (jovens-adultos) e 20 indivíduos de 31 a 50 anos (adultos), para realização de avaliação comportamental (Teste de reconhecimento de sentença na presença de ruído estável e modulado) e eletrofisiológica (Potencial Evocado Auditivo Cortical) para investigação do BMM. Os resultados foram analisados através do Test-t pareado e ANOVA para medidas repetidas, seguido pelo teste post-hoc de Bonferroni (p-valor <0,05).

**Resultados:**

Foi identificada uma menor interferência do ruído modulado nas medidas de latência e amplitude dos componentes corticais, gerando uma redução significativa na latência de P1 e aumento da amplitude de P2 em ambos os grupos de participantes. O ruído estável gerou limiares eletrofisiológicos e comportamentais mais elevados comparado ao ruído modulado. Uma maior magnitude do BMM foi observada no grupo jovem-adulto.

**Conclusão:**

Identificou-se em ambos os grupos de participantes uma menor interferência do ruído modulado no tempo de codificação da resposta auditiva neural e no processo de discriminação neural da fala. Além disso, os limiares comportamentais e eletrofisiológicos foram tipicamente mais elevados diante do ruído estável quando comparado ao ruído modulado, apontando para uma correspondência da medida do BMM entre os domínios da audição. A magnitude do BMM superior no grupo de jovens-adultos, especialmente no domínio eletrofisiológico, sugere uma maior habilidade de resolução temporal em indivíduos mais jovens.

## INTRODUÇÃO

A utilização dos Potenciais Evocados Auditivos de Longa Latência (PEALL), como o Potencial Evocado Auditivo Cortical (PEAC) tem crescido significativamente na prática audiológica clínica, para diagnóstico e acompanhamento auditivo, como também nas pesquisas, para desenvolvimento de novos estudos e metodologias relacionadas ao Processamento Auditivo Central (PAC) em diferentes populações^(1)^.

O PEAC possibilita a avaliação da atividade neuroelétrica do córtex auditivo, fornecendo dados biológicos importantes do processamento auditivo humano, podendo ser facilmente aplicado, uma vez que independe da resposta, atenção e interação do paciente, sendo realizado sob níveis mínimos de desconforto^(2)^.

Uma das habilidades do PAC que pode ser estudada por meio do PEAC, consiste na capacidade de Processamento Auditivo Temporal (PAT) do indivíduo, que integra funções importantes na comunicação, especialmente na compreensão da fala^(3)^. O PAT corresponde a decodificação dos aspectos temporais da audição, permitindo que o indivíduo processe alterações mínimas em som/fala dentro de um período, discriminando características físicas de tempo, frequência e intensidade do sinal acústico^(4)^.

O fenômeno conhecido como *Masking Release,* utilizado em português como Benefício do Mascaramento Modulado – BMM^(5)^, consiste em um efeito relacionado ao PAT que corresponde à identificação de sinais audíveis de um som/fala diante de oscilações de um ruído de fundo simultâneo^(6)^. Em outras palavras, o BMM é ocasionado quando flutuações temporais do ruído mascarante possibilitam sinais audíveis do som/fala alvo, provocando consequentemente, melhora no desempenho de reconhecimento do sinal alvo^(7)^.

Medidas comportamentais da audição, por meio de testes psicoacústicos, foram inicialmente utilizadas para investigação do BMM^(5,8-10)^, constatando melhora no limiar de reconhecimento de fala diante do mascaramento modulado, quando comparado ao mascaramento estável/constante, e associando este desempenho à capacidade de processamento temporal do indivíduo.

Pesquisas com interesse em investigar o BMM no domínio da eletrofisiologia da audição e estudar o efeito da modulação do mascaramento no comportamento de medidas objetivas corticais, como o PEAC, têm apontado que tais testes possuem o potencial de avaliar habilidades do PAT; além de apresentar resultados objetivos na avaliação de indivíduos incapazes de fornecer respostas comportamentais confiáveis^(6,11-13)^.

O estudo do fenômeno do BMM nos domínios eletrofisiológico e comportamental tem possibilitado o aprimoramento de pesquisas a respeito das habilidades do PAT^(6)^ e o estabelecimento de parâmetros de análise do reconhecimento da fala em situações de ruído^(13)^. No entanto, é observado na literatura que, geralmente, os estudos que utilizaram medidas eletrofisiológicas na investigação do BMM, não realizaram testes comportamentais em paralelo^(11,12)^.

Presume-se que, com que a realização de medidas eletrofisiológicas paralelas a medidas comportamentais seja possível determinar se tais medidas objetivas são preditivas do desempenho comportamental auditivo no estudo do BMM e de seu efeito no processamento temporal da audição. À vista disso, este estudo tem como objetivo analisar o BMM em medidas eletrofisiológica e comportamental em jovens e adultos normouvintes.

## MÉTODO

A pesquisa trata-se de um estudo analítico, do tipo observacional e transversal, realizado no Laboratório de Audiologia do Departamento de Fonoaudiologia da Universidade Federal de Pernambuco (UFPE) no período de agosto de 2022 a junho de 2023. O protocolo desta pesquisa é baseado na Resolução nº 466/2012 do Conselho Nacional de Saúde - CNS para estudos com seres humanos e foi aprovado pelo Comitê de Ética e Pesquisa em Seres Humanos da UFPE, com o parecer de número 5.140.668.

Participaram inicialmente desta pesquisa, 22 indivíduos de 18 a 30 anos (jovens-adultos) e 23 indivíduos de 31 a 50 anos (adultos) recrutados no campus universitário, após divulgação da pesquisa por meios eletrônicos. Todos os participantes foram orientados quanto ao objetivo e procedimentos necessários para realização do estudo. Após aceitarem participar da pesquisa, foi assinado o Termo de Consentimento Livre e Esclarecido - TCLE em duas vias.

Quanto aos critérios de elegibilidade para participação da pesquisa, foram considerados como critérios de inclusão, indivíduos entre 18 e 50 anos de idade sem perda auditiva, sendo excluídos indivíduos com histórico de doenças neurológicas e/ou psiquiátricas, déficits cognitivos, com malformações de pavilhão auricular e meato acústico externo, além de perda auditiva de qualquer tipo e/ou grau. Para garantir os critérios de seleção da pesquisa, foram realizados em data previamente agendada, anamnese detalhada com informações de saúde geral e auditiva dos participantes, além da realização de exames audiológicos básicos (inspeção do meato acústico externo, audiometria e imitanciometria) e teste para triagem cognitiva (Teste de Avaliação Cognitiva Montreal – MoCA)^(14)^.

A existência de alterações e/ou malformações na orelha externa e/ou média foi descartada com a inspeção do meato acústico externo, além do exame de imitanciometria (sonda de 226 Hz), sendo considerado como normalidade a presença de curva timpanométrica tipo A com reflexos ipsi e contralaterais presentes^(15,16)^. Para confirmação de normalidade dos limiares auditivos tonais, no exame de audiometria foi considerado como padrão de normalidade a média quadritonal das frequências de 500 Hz, 1000 Hz, 2000 Hz e 4000 Hz inferior a 20 dB NA, em ambas as orelhas^(17)^. Para a triagem cognitiva (MoCA), o resultado de normalidade foi obtido com pontuação ≥ 26 pontos, como sugerido no próprio teste^(14)^.

Após a realização dos exames prévios e exclusão dos participantes que não atendiam aos critérios de seleção da pesquisa, a amostra final foi composta por 40 indivíduos que foram submetidos à avaliação comportamental e eletrofisiológica, de modo aleatório, para investigação do BMM.

### Avaliação comportamental

Para investigação do BMM no domínio comportamental, os participantes foram submetidos ao teste de reconhecimento de sentença na presença de ruído estável e modulado, utilizando as 12 listas (com 20 sentenças cada) do teste HINT-Brasil, gravadas na voz masculina de um locutor brasileiro nativo^(18)^. O teste foi realizado para obtenção do Limiar de Reconhecimento de Sentenças (LRS) nas duas condições de ruído, sendo considerado como limiar comportamental do indivíduo. O ruído estável foi apresentado a 65 dB NPS e o ruído modulado variou entre as intensidades de 65 e 30 dB NPS com taxa de modulação de 10 Hz^(8)^. O ruído utilizado (speech-shaped noise - SSN) possui o envelope de espectro de frequências semelhante ao envelope do espectro de frequências das sentenças utilizadas no teste; o ruído modulado foi produzido por meio da modificação do ruído estável com a utilização do processador acústico de sinal *Tucker-Davis Technologies*-RX6 (TDT-RX6)^(5,8)^. As apresentações dos ruídos mascarantes seguiram uma ordem aleatória em cada indivíduo.

Para realização do teste, os participantes foram posicionados em poltrona dentro de uma cabina acústica e orientados a repetir as sentenças na presença do ruído de fundo competitivo exatamente como escutaram. O monitoramento e registro das respostas dos participantes foram realizados de maneira simultânea pelo examinador posicionado fora da cabina, e com utilização do software *Matlab* (*Matrix Laboratory*®), versão R2012a. As sentenças e o ruído de fundo competitivo foram enviados (via TDT-RX6) e apresentados de maneira monoaural, por meio de fone auditivo supra-aural (*Sennheiser* HD580), à orelha direita. Cada sentença do HINT-Brasil foi apresentada uma única vez para o mesmo participante, a fim de eliminar o viés de aprendizagem. Além disso, a escolha das listas ocorreu de forma aleatória. A duração do teste foi de aproximadamente 40 minutos, sendo realizadas pausas mediante solicitação dos sujeitos.

A resposta correta para cada sentença foi considerada a partir de sua repetição exata, sendo considerado como erro, qualquer alteração na utilização de artigos, conjugação verbal e inclusão ou omissões de palavras. Considerando a importância da reprodutibilidade na obtenção do LRS, foram realizadas três medições da pesquisa de limiar para as duas condições de ruído. O LRS final de cada participante (para os dois tipos de ruído) foi obtido por meio da média das três medições do limiar em dB NPS. O BMM foi definido através da diferença entre o LRS em presença de ruído estável (tomado como referência) e o LRS em presença de ruído modulado.

Para a pesquisa do LRS, a intensidade inicial utilizada foi superior ao limiar de reconhecimento esperado, sendo 60 dB NPS na condição de ruído modulado e 70 dB NPS na condição do ruído estável. O método de obtenção do LRS, pelo software *Matlab*, consistia no tipo descendente-ascendente transformado (*two down - one up*)^(19)^, onde a cada duas respostas corretas consecutivas, a intensidade do sinal diminuiu em 2 dB na sentença seguinte, e a cada resposta incorreta, a intensidade de apresentação da sentença seguinte foi aumentada em 2 dB. A obtenção de cada LRS se deu após seis reversões (aumento ou diminuição da intensidade das sentenças apresentadas), por meio do cálculo da média dos quatro níveis (intensidades) finais de reversão.

### Avaliação eletrofisiológica

Para investigação do BMM no domínio eletrofisiológico, os participantes foram submetidos ao PEAC (*Intelligent Hearing Systems* – IHS), com estímulo de fala sintética /ba/ na presença de ruído de fala (SSN)^(20)^, apresentados de forma simultânea, sendo o ruído em duas condições (estável e modulado). O estímulo /ba/ foi apresentado em forma de onda modificada (taxa de 24.414 Hz) para ser compatível com o sinal digital da plataforma de processamento *Tucker-Davis Technologies*-RX6 (TDT-RX6) e calibrado com referência ao dB NPS de um tom contínuo de 1kHz, pico equivalente (dB NPSpe). Para registro dos potenciais, um sistema de gravação foi sincronizado entre o *Smart* EP do IHS com o TDT- RX6 por meio de um marcador de tempo-evento (“*Trigger*”) coincidente com o início de cada estímulo /ba/. O estímulo de fala /ba/ e o ruído foram apresentados de forma monoaural para a orelha direita via fone de inserção (ER2) eletromagneticamente blindado, ligados diretamente do TDT-RX6. O /ba/ teve duração de 80 milissegundos (ms) e foi apresentado a uma intensidade fixa de 65 dB NPSpe e taxa de 3,8 estímulos por segundo.

Para aquisição do PEAC, o ruído foi apresentado simultaneamente ao estímulo /ba/ em três condições distintas: a) /ba/ e ruído estável com intensidade de 30 dB NPSpe (ruído estável fraco); b) /ba/ e ruído estável com intensidade de 65 dB NPSpe (ruído estável forte); c) /ba/ e ruído modulado em 25 Hz nas intensidades de 30 e 65 dB NPSpe (Figura 1). A apresentação das diferentes condições de ruído foi realizada de maneira aleatória em cada indivíduo.

**Figura 1:** Ilustração do estímulo de fala diante das três condições de mascaramento

Legenda: Representação do estímulo de fala /ba/ (onda na cor preta) diante das três condições de ruído utilizadas no teste eletrofisiológico

Os participantes foram posicionados em poltrona reclinável dentro de cabina acústica, assistindo a um vídeo sem áudio e orientados a não dormir durante a realização do exame. A limpeza da pele com álcool 70% e gel abrasivo (NuPrep®) antecedeu a colocação dos eletrodos, que foram posicionados nas seguintes configurações: dois eletrodos de referência de polaridade negativa posicionados na região dos lóbulos direito (A1) e esquerdo (A2); um eletrodo de polaridade positiva colocado no vértice da cabeça (Cz) e um eletrodo terra posicionado na região inferior da fronte (Fpz). As olivas utilizadas nos fones de inserção foram descartáveis. A duração total do exame foi de aproximadamente uma hora em cada participante, sendo realizadas pausas mediante solicitação dos sujeitos.

Os potenciais corticais (P1, N1 e P2) foram analisados quanto a sua latência (em milissegundos-ms), amplitude (em microvolts-µV) e morfologia, diante das três condições de ruído. Todos os registros dos traçados do PEAC foram analisados individualmente, de maneira cega, por três avaliadores com experiência em eletrofisiologia, para identificação e marcação dos potenciais. O componente P1 foi considerado como a primeira onda cortical positiva mais robusta em torno de 50ms, o componente N1 foi analisado como o vale subsequente à onda P1, com maior negatividade, e a resposta de P2 foi identificada como a onda positiva mais robusta após N1.

O limiar eletrofisiológico no PEAC também foi investigado em cada participante, eliciado pelo estímulo de fala /ba/ diante das condições de ruído estável forte e ruído modulado. A pesquisa do limiar foi realizada a partir da diminuição da intensidade do estímulo de fala de 10 em 10 dB até o desaparecimento do complexo P1-N1-P2 e em seguida o aumento de 5 em 5 dB até o seu aparecimento. A partir da obtenção do limiar eletrofisiológico, a magnitude do BMM foi mensurada para cada sujeito, diante da diferença em decibéis (dB NPSpe) entre as condições de mascaramento estável e modulado.

### Análise estatística dos dados

A análise estatística foi realizada por meio do programa *Statistical Package for the Social Sciences* (SPSS) versão 20.0. Os resultados foram expressos através das medidas estatísticas de média, mediana, desvio-padrão e intervalo de confiança (95%), em apresentação tabular. A normalidade das amostras por grupo foi verificada com o teste de *Shapiro-Wilk* e utilização do critério de Hair et al.^(21)^ para análise de assimetria (- 2 e +2) e curtose (-7 e +7), sendo observada uma distribuição normal dos dados. Para análise inferencial dos componentes corticais em cada grupo de ruído utilizou-se ANOVA para medidas repetidas, seguido pelo teste *post-hoc* de *Bonferroni* (p-valor <0,05). O *Test-t* pareado foi utilizado para análise dos limiares eletrofisiológicos e comportamental entre os dois grupos de ruído, estável e modulado (p-valor <0,05).

## RESULTADOS

Um total de 45 indivíduos foram recrutados inicialmente para a pesquisa, onde 22 participantes possuíam idades de 18 a 30 anos e 23 participantes com idades de 31 a 50 anos. Cinco participantes foram excluídos da amostra final, onde dois apresentaram oclusão do meato acústico externo, um apresentou limiares compatíveis com perda auditiva leve e dois participantes não compareceram ao exame comportamental e eletrofisiológico na data agendada. Com isso, 40 participantes com audição dentro dos critérios de normalidades pré-estabelecidos compuseram a amostra final do estudo. Os participantes foram divididos em dois grupos de acordo com a idade, sendo o grupo “Jovem-adulto” com faixa etária de 18 a 30 anos e o grupo “Adulto”, de 31 a 50 anos de idade. O grupo Jovem-adulto foi composto por 20 participantes (15 mulheres e 5 homens), com média de idade de 22,8 anos e o grupo Adulto foi composto por 20 participantes (10 mulheres e 10 homens), com média de idade de 37,7 anos.

A resposta média geral (*grand average*) das ondas do PEAC para cada tipo de ruído mascarante e entre os dois grupos de participantes é mostrada nos painéis A – F da Figura 2. Os traços individuais são mostrados como linhas na cor cinza claro e as respostas médias de cada grupo como linhas escuras pesadas. É possível observar uma semelhança nas respostas médias das ondas corticais entre os grupos etários. As ondas corticais na condição do ruído estável forte são demonstradas com menor amplitude, sobretudo no componente P2 (representado pelo segundo pico positivo) e no componente N1 (representado pelo primeiro vale negativo) para ambos os grupos de participantes (Figura 2B e E).

**Figura 2:** Grandes médias das ondas do PEAC para as três condições de mascaramento e em cada grupo de participantes.

A Tabela 1 descreve, para cada grupo etário, os valores de latência dos componentes corticais (P1, N1, P2) evocados pelo estímulo de fala /ba/ diante dos três diferentes tipos de mascaramento apresentados e a comparação entre as médias obtidas. É possível observar que tanto para o grupo jovem-adulto quanto para o grupo adulto, os valores de latência diante do mascaramento com ruído modulado foram menores quando comparado ao ruído estável forte.

**Tabela 1:** Comparação das médias de latência dos componentes P1, N1 e P2 entre as diferentes condições de ruído

**Latência**	**Ruído estável fraco**	**Ruído estável forte**	**Ruído modulado**	**ANOVA**
**(ms)**	**Média ± DP**	**Média ± DP**	**Média ± DP**	**Post-hoc (Bonferroni)**
	**(IC _95%_)**	**(IC _95%_)**	**(IC _95%_)**	
**Jovem-adulto**				
Componente	56,3 ± 11,5	68,9 ± 20,1	51,9 ± 8,0	**p 0,016 ^(a)^** *
				p 0,896 ^(b)^
P1	(50,8 - 61,7)	(59,4 - 78,3)	(48,1 - 55,6)	**p 0,003 ^(c)^** *
Componente	109,6 ± 10,0	118,7 ± 23,2	116,6 ± 12,3	p 0,328 ^(a)^
N1	(104,9 - 114,3)	(107,8 - 129,6)	(110,8 - 122,4)	p 0,059 ^(b)^
				p 1,000 ^(c)^
				
Componente	165,9 ± 16,1	174,9 ± 24,2	168,3 ± 12,9	p 0,521 ^(a)^
P2	(158,3 - 173,4)	(163,1 - 186,2)	(162,2 - 174,4)	p 1,000 ^(b)^
				p 0,488 ^(c)^
**Adulto**				
Componente	56,4 ± 9,3	65,6 ± 10,3	54,8 ± 7,5	**p 0,024 ^(a)^** *
				p 1,000 ^(b)^
P1	(52,1 - 60,8)	(60,7 - 70,5)	(51,3 - 58,4)	**p 0,002 ^(c)^** *
Componente	104,6 ± 7,7	119,9 ± 21,5	116,7 ± 15,1	p 0,022 ^(a)^
				p 0,008 ^(b)^
N1	(100,9 - 108,2)	(109,8 - 129,9)	(109,6 - 123,8)	p 1,000 ^(c)^
Componente	163,8 ± 13,7	178,7 ± 27,4	173,2 ± 22,0	p 0,108 (a)
				p 0,204 (b)
P2	(157,4 - 170,3)	(165,9 - 191,6)	(162,8 - 183,4)	p 0,793 (c)

* Diferença estatisticamente significativa

^(a)^Comparação das médias entre os ruídos estável fraco e forte;

^(b)^Comparação das médias entre os ruídos estável fraco e ruído modulado;

^(c)^Comparação das médias entre os ruídos estável forte e ruído modulado

Legenda: ms = milissegundos; DP = Desvio Padrão; IC _95%_ = Intervalo de Confiança de 95%

No teste de comparação das medidas de latência (ANOVA, *post-hoc Bonferroni*) constatou-se que a redução dessa medida com a modulação do ruído foi estatisticamente significativa (p<0,05) na resposta cortical de P1 em relação à condição de ruído estável forte, para ambos os grupos de participantes. Não houve diferença significativa nas latências, ou seja, no tempo de codificação neural entre o mascaramento modulado e o estável fraco.

Na Tabela 2, estão descritos, para os dois grupos de participantes, os valores de amplitude das respostas corticais (P1, N1, P2) diante das três condições de mascaramento utilizadas, bem como a comparação entre as médias obtidas. Foi possível observar que no grupo de jovens-adultos, os valores das médias de amplitude dos componentes corticais foram maiores diante do mascaramento com ruído modulado quando comparado ao ruído estável forte.

**Tabela 2:** Comparação das médias de amplitude dos componentes P1, N1 e P2 entre as diferentes condições de ruído

**Amplitude**	**Ruído estável fraco**	**Ruído estável forte**	**Ruído modulado**	**ANOVA**
**(µV)**	**Média ± DP**	**Média ± DP**	**Média ± DP**	**Post-hoc (Bonferroni)**
	**(IC _95%_)**	**(IC _95%_)**	**(IC _95%_)**	
		**Jovem-adulto**		
Componente				
P1	5,7 ± 1,9	5,2 ± 1,9	5,8 ± 1,6	p 0,565 (a)
	(4,8 - 6,6)	(4,3 - 6,1)	(5,0 - 6,6)	p 1,000 (b)
				p 0,373 (c)
				
Componente	5,1 ± 3,1	4,2 ± 3,2	4,4 ± 2,4	p 0,123 ^(a)^
N1	(3,6 - 6,6)	(2,6 - 5,7)	(3,2 - 5,5)	p 0,370 ^(b)^
				p 1,000 ^(c)^
				
Componente	5,0 ± 4,1	3,3 ± 2,5	5,4 ± 2,4	**p 0,027 ^(a)^** *
P2	(3,0 - 6,9)	(2,1 - 4,5)	(4,2 - 6,5)	p 1,000 ^(b)^
				p 0,001 ^(c)^ *
		**Adulto**		
				
Componente	5,3 ± 1,8	5,4 ± 2,0	5,4 ± 1,7	p 1,000 ^(b)^
P1	(4,4 - 6,2)	(4,4 - 6,3)	(4,6 - 6,3)	p 1,000 ^(b)^
				p 1,000 ^(b)^
				
Componente	5,4 ± 2,3	4,1 ± 3,1	4,8 ± 2,4	p 0,055 ^(a)^
N1	(4,3 - 6,5)	(2,6 - 5,6)	(3,6 - 5,9)	p 0,368 ^(b)^
				p 0,690 ^(c)^
				
Componente	4,9 ± 2,6	3,4 ± 2,0	5,2 ± 1,8	p 0,084 ^(a)^
P2	(3,6 - 6,1)	(2,5 - 4,4)	(4,4 - 6,1)	p 1,000 ^(b)^
				p 0,006 ^(c)^ *

* Diferença estatisticamente significativa

^(a)^Comparação das médias entre os ruídos estável fraco e forte;

^(b)^Comparação das médias entre os ruídos estável fraco e ruído modulado;

^(c)^Comparação das médias entre os ruídos estável forte e ruído modulado

Legenda: µV = microvolts; DP = Desvio Padrão; IC _95%_ = Intervalo de Confiança de 95%

O teste comparativo (ANOVA, *post-hoc Bonferroni*) demonstrou que o aumento das medidas de amplitude na condição de modulação do ruído foi estatisticamente significativo (p<0,05) na resposta cortical de P2 em relação à condição de ruído estável forte. No grupo adulto, as respostas de amplitude foram maiores nos componentes N1 e P2 diante do mascaramento com ruído modulado quando comparado ao ruído estável forte, com diferença estatisticamente significativa (p<0,05) apenas em P2, semelhante ao grupo mais jovem. Assim como nas respostas de latências, não houveram diferenças significativas nas amplitudes, ou seja, na magnitude da resposta neural entre o mascaramento modulado e o estável fraco, para ambas as faixas etárias.

Na pesquisa dos limiares eletrofisiológicos e comportamentais, os resultados expressos na Tabela 3 evidenciam, para os dois grupos de participantes, que a condição de modulação do mascaramento resultou em limiares estatisticamente inferiores (p<0,05; *teste t* pareado) quando comparado ao mascaramento estável em ambos os domínios da audição.

**Tabela 3:** Descrição do BMM e limiares eletrofisiológico e comportamental diante do de ruído estável ruído modulado

	**Ruído estável**	**Ruído modulado**	**BMM**	**Test t pareado**
**(dB NPS)**	**Média ± DP**	**Média ± DP**	**Média ± DP**	**p - valor**
	**(IC _95%_)**	**(IC _95%_)**	**(IC _95%_)**	
		
***Jovem-adulto***				
				
**Limiar eletrofisiológico**	49,7 ± 6,3	41,2 ± 5,3	9,5 ± 4,6	p 0,000*
	(46,7 - 52,7)	(38,7 - 43,7)	(7,0 - 11,4)	
				
**Limiar comportamental**	59,1 ± 1,0	50,3 ± 1,8	8,7 ± 1,4	**p 0,000** *
	(58,6 - 59,6)	(49,4 - 51,2)	(8,1 - 9,4)	
***Adulto***				
				
**Limiar eletrofisiológico**	52,5 ± 4,1	45,5 ± 3,5	6,7 ± 2,4	**p 0,000** *
	(50,5 - 54,4)	(43,8 - 47,1)	(5,6 - 7,9)	
				
**Limiar comportamental**	59,2 ± 1,0	51,0 ± 1,6	8,1 ± 1,3	**p 0,000** *
	(58,7 - 59,6)	(50,2 - 51,8)	(7,5 - 8,7)	

* Diferença estatisticamente significativa

Legenda: DP = Desvio Padrão; IC _95%_ = Intervalo de Confiança de 95%; dB NPS = decibel nível de pressão sonora; BMM = Benefício do Mascaramento Modulado

Este benefício, observado pela redução dos limiares diante da modulação do ruído, é expresso pela medida de BMM, sendo maior no grupo de jovens-adultos em ambos os domínios da audição, como ilustrado na Figura 3.

**Figura 3:** Benefício do mascaramento modulado (BMM) eletrofisiológico e comportamental para os dois grupos de participantes

Legenda: PEAC individuais (cinza claro) e médios de grupo (preto) para Ruído Estável Fraco, Ruído Estável Forte e Ruído Modulado, para cada grupo de participantes

## DISCUSSÃO

O estudo do efeito do ruído modulado diante da detecção do estímulo de fala por meio do PEAC permite a análise do benefício da modulação e seu efeito no processamento temporal da audição de maneira objetiva, sendo válido considerar a importância da avaliação psicoacústica a fim de investigar uma predição deste efeito no desempenho comportamental auditivo. Em virtude disso, este estudo avaliou o efeito da modulação do ruído mascarante com estímulo de fala por meio do paralelo entre os domínios eletrofisiológico e comportamental em jovens e adultos normouvintes.

O complexo cortical P1-N1-P2 evocado pelo estímulo de fala /ba/ mascarado pelos ruídos estável e modulado permitiu a análise das respostas corticais referentes às características acústicas do processamento sonoro a nível talâmico, córtex auditivo primário e áreas de associação^(22)^. A aplicação do PEAC com estímulos complexos e em condições de mascaramento favorece o estudo do BMM, principalmente pela proximidade dos geradores de seus potenciais com a percepção do som^(23)^.

Desta maneira, as respostas de latência e amplitude dos componentes corticais analisados podem refletir a percepção das características acústicas da fala e determinar a integridade da codificação neural de cada indivíduo, além de inferir sobre a influência do ruído no tempo de percepção da fala e sobre a magnitude da atividade cortical no processamento desses sinais complexos^(24)^.

Com base na análise das medidas de latência, foi possível observar no presente estudo uma maior interferência do ruído estável forte no sincronismo neural para geração dos potenciais corticais (P1-N1-P2) em ambos os grupos de participantes, resultando em latências maiores nesta condição de mascaramento (Tabela 1). Uma menor interferência neste sincronismo neural, semelhante nos dois grupos de idade, foi estatisticamente constatada na geração do componente P1 diante da diminuição da intensidade do ruído (ruído estável fraco) e de sua modulação. Este achado demonstra que apesar da perturbação do ruído na percepção da fala, o ruído modulado possui uma menor interferência no tempo de codificação da resposta auditiva neural (representado por P1), quando comparado ao ruído estável forte, não havendo diferença no tempo de codificação neural entre o mascaramento modulado e o estável fraco.

Apesar do atraso esperado nas latências das respostas corticais em condições de ruído, estudos também apontam uma menor interferência no tempo de processamento neural quando este ruído apresenta modulações, resultando em uma diminuição sistemática nas respostas de latência em comparação ao ruído estável^(6,12,13)^. A menor interferência significativa da modulação expressa no componente P1 pode ser explicada pelo fato deste potencial estar relacionado à detecção e codificação das características de frequência e tempo do estímulo acústico^(25)^, sendo estas propriedades mais bem exploradas durante a redução dos níveis de mascaramento do ruído modulado, facilitando o tempo de codificação do sinal da fala^(7)^.

A respeito das amplitudes (Tabela 2), os resultados evidenciaram, semelhantemente aos resultados de latência, uma menor interferência do mascaramento modulado nessas respostas, resultando em amplitudes maiores quando comparado à condição de ruído estável forte, especialmente para o componente P2 (p<0,05), para ambos os grupos de participantes. A morfologia das ondas do *grand average* confirma a maior interferência do ruído estável forte nas respostas corticais, expressa pelas menores amplitudes da segunda onda positiva (P2) e do primeiro vale negativo (N1) (Figura 2B e E). Estes resultados corroboram estudos que apontam a diminuição da relação sinal/ruído gerada pela modulação na intensidade do ruído mascarante como resultado de uma menor interferência deste mascaramento na magnitude das respostas neurais expressas pelas amplitudes dos potenciais^(6,26)^.

A menor interferência significativa gerada pelo ruído modulado na amplitude de P2 demonstra que apesar do mascaramento, a condição de modulação ainda permite uma melhor mobilização de neurônios no processo de discriminação neural do sinal de fala, sofrendo maior perturbação na condição de ruído estável^(27)^. Em relação às medidas de latência e amplitude dos componentes corticais estudados, não houve diferenças consideráveis nas respostas em ambos os grupos de participantes, apontando para um desempenho auditivo cortical semelhante nas faixas etárias estudadas.

Os resultados da pesquisa de limiar eletrofisiológico e comportamental para ambos os grupos de idade (Tabela 3) corroboram achados na literatura que inferem que o ruído estável possui maior efeito mascarante nos limiares mínimos de detecção de um sinal sonoro, gerando limiares tipicamente mais elevados quando comparado ao ruído modulado em ambos os domínios da audição^(5,6,10)^. Embora os limiares médios do PEAC tenham sido inferiores aos registrados nos limiares comportamentais, a diferença foi inferior a 10 dB, o que corresponde com outros achados na literatura que observaram limiares corticais comparativamente inferiores à limiares comportamentais em até 10 dB^(28)^.

Na presente pesquisa, os limiares estatisticamente menores diante do ruído modulado em ambos os domínios da audição demonstram que a realização do PEAC pode ser preditiva na avaliação do desempenho comportamental auditivo, confirmando o benefício da modulação do ruído na detecção e percepção do estímulo de fala. O estudo entre limiares eletrofisiológicos e comportamentais tem sido apontada em outras pesquisas, mostrando uma correlação entre essas medidas^(23,29)^, onde o PEAC tem sido visto com potencial de avaliar as habilidades de processamento temporal e oferecer resultados equivalentes confiáveis de limiares de detecção de fala diante de ruídos estáveis e modulados^(6,13)^.

A diferença do limiar entre as duas condições de mascaramento (estável e modulado), interpretada como a mensuração do BMM, foi comparavelmente similar entre os domínios eletrofisiológico e comportamental, em ambos os grupos de participantes (Tabela 3), apontando para uma correspondência dessa medida entre os domínios da audição. O valor do BMM, especialmente no domínio eletrofisiológico, foi superior no grupo jovem-adulto (9,5 dB) em relação ao grupo adulto (6,7 dB), demonstrando um melhor aproveitamento da modulação do ruído em indivíduos mais jovens (Figura 3), sugerindo que indivíduos jovens possuem uma maior habilidade do sistema auditivo em perceber as pistas acústicas da fala que não estão mascaradas nos espaços de tempo de menor intensidade do ruído, em virtude da modulação^(5,30)^.

A magnitude do BMM no domínio comportamental foi aproximada entre os grupos de participantes, demonstrando pouca variação relacionada à faixa etária estudada no teste psicoacústico. Estudos de investigação do BMM com utilização de teste comportamentais em jovens e adultos, constaram magnitude de 8,6 dB e 7,3 dB, semelhantes aos resultados encontrados na presente pesquisa^(5,8)^.

Pesquisas relacionadas ao estudo do efeito da modulação do ruído no sistema auditivo sugerem que a resposta do BMM pode ser adotada como uma medida representativa da capacidade de resolução temporal^(6,31)^, uma vez que esta habilidade se refere à identificação de curtos períodos temporais diante de dois sinais acústicos^(32)^. Desta maneira, é possível considerar que a presença do efeito do BMM esteja relacionada à integridade do processamento temporal da audição em indivíduos normouvintes.

Os resultados desta pesquisa permitiram a análise do BMM nos domínios comportamental e eletrofisiológico da audição, possibilitando a compreensão do efeito da modulação do ruído no processamento auditivo temporal, bem como sua relação com a habilidade de resolução temporal. Apesar da presente pesquisa ser restrita à população jovem e adulta normouvinte, a realização de mais estudos com essa faixa etária e até mesmo com a população idosa, com e sem perda auditiva, poderão contribuir para a melhor compreensão do BMM.

## CONCLUSÃO

Os achados da presente pesquisa apontaram, em ambos os grupos de participantes, uma menor interferência do ruído modulado no tempo de codificação da resposta auditiva neural (redução significativa da latência de P1) e uma menor perturbação do ruído modulado no processo de discriminação neural da fala (aumento significativo nas amplitudes de P2). As pesquisas dos limiares comportamental e eletrofisiológico evidenciaram que o ruído estável gerou limiares tipicamente mais elevados comparado ao ruído modulado em ambos os testes, apontando para uma correspondência da medida do BMM entre os domínios da audição. Os jovens-adultos apresentaram uma magnitude do BMM superior aos adultos, especialmente no domínio eletrofisiológico, o que sugere uma maior habilidade de resolução temporal em indivíduos mais jovens.
